# A case of incorrect evaluation of intestinal patency by early dissolution of a patency capsule

**DOI:** 10.1002/deo2.288

**Published:** 2023-08-24

**Authors:** Teppei Omori, Toshifumi Hara, Shun Murasugi, Harutaka Kambayashi, Yu Sasaki, Miki Koroku, Maria Yonezawa, Keiichi Morishita, Shinichi Nakamura, Katsutoshi Tokushige

**Affiliations:** ^1^ Institute of Gastroenterology Tokyo Women's Medical University Tokyo Japan; ^2^ Department of Gastroenterology Hachioji Digestive Disease Hospital Tokyo Japan

**Keywords:** capsule endoscopy, patency capsule, radiography, stenosis, small intestine

## Abstract

A 60‐year‐old man presented with a suspected small intestinal tumor on positron‐emission tomography‐computed tomography. Small bowel capsule endoscopy (SBCE) was planned for close examination of the small intestine. To avoid retention of the SBCE due to strictures, a patency capsule (PC) was first used to evaluate patency. However, PC discharge was not visually confirmed during the 24‐h period. No obvious PC was observed on plain abdominal radiography performed in the standing position. The patient underwent SBCE, assuming that the PC had been shed inconspicuously. SBCE revealed a neoplastic lesion with stenosis at a site thought to be the upper small intestine and remained stagnant at the same site for the duration of the battery. In addition, in the SBCE image, a PC shell was captured in the intestinal tract on the oral side of the stenosis. When the pre‐SBCE plain abdominal radiograph was enlarged to confirm the details, PC was observed in the lateral and decubitus views as a dissolved shell only. To the best of our knowledge, no previous report has described the complete dissolution of a PC leaving only its shell during a 30‐hour patency evaluation period. This case illustrates that, in the absence of visual confirmation of a PC discharge, PC may have remained in the body due to premature dissolution. Additional examinations or plain X‐ray imaging should be performed to confirm this, with no preconceived notions that the PC will not dissolve within 30 hours of administration.

## INTRODUCTION

Small bowel capsule endoscopy (SBCE) is a minimally invasive technique that allows observation of the entire small bowel but carries aspiration and retention risks.^1^ When examining for Crohn's disease and other potentially stenotic conditions, gastrointestinal tract patency should ideally be evaluated with a PillCam patency capsule (PC), a time‐dependent dissolvable capsule, to evaluate the risk of capsule endoscope retention in stenotic areas.^2^

Here, we report a case of an adverse event with a PC, in which the capsule dissolved earlier than designed, resulting in an inaccurate assessment of patency and SBCE retention in the stenotic small bowel.

## CASE PRESENTATION

**FIGURE 1:**
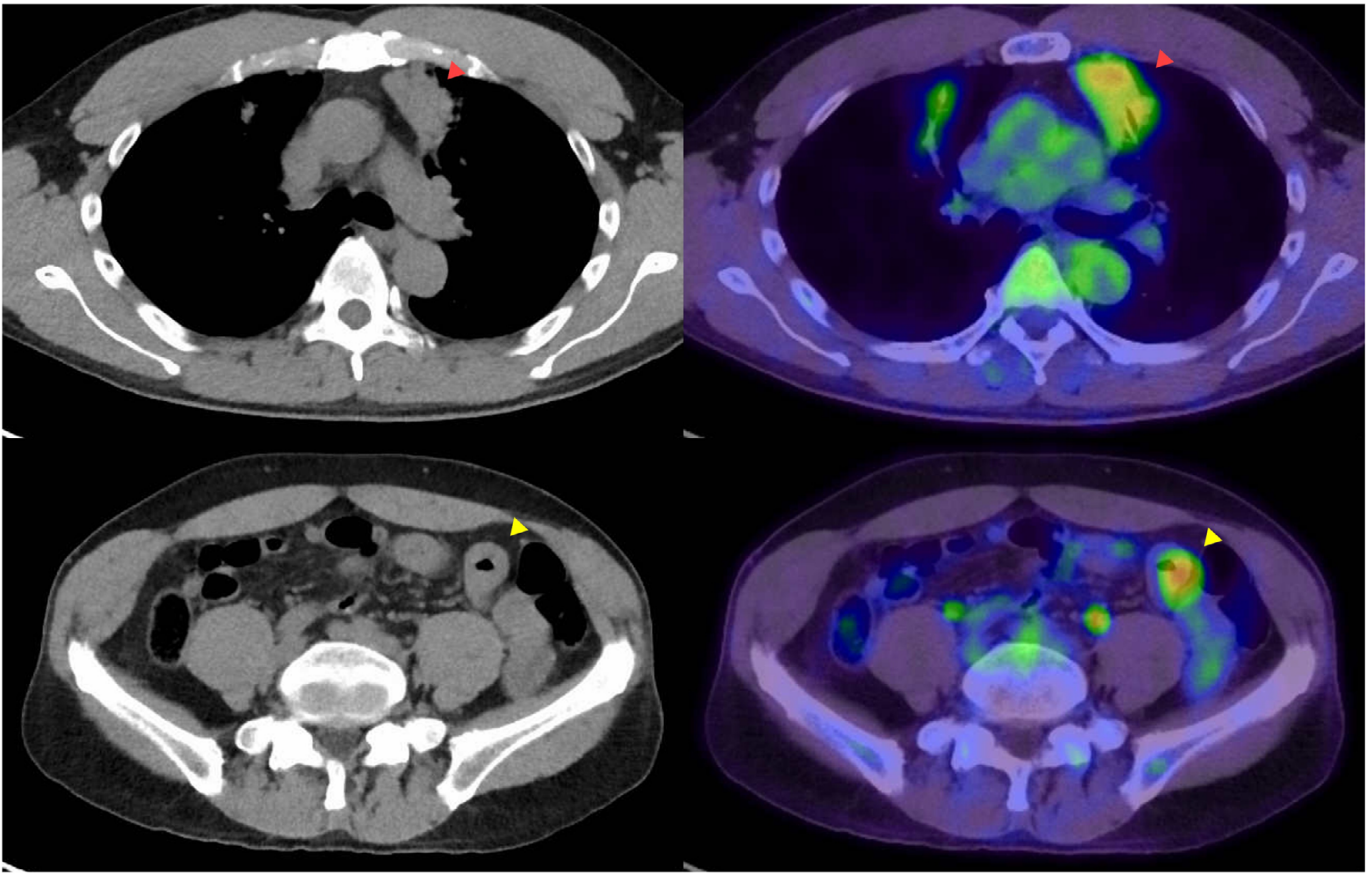
^18^F‐fluorodeoxyglucose‐positron emission tomography‐computed tomography (^18^FDG‐PET‐CT) and non‐contrast CT. Upper row: lung lesion (red arrowheads), lower row: small intestinal lesion with wall thickening in the left lower abdominopelvic cavity (yellow arrowheads).

A 60‐year‐old man was diagnosed with mucosa‐associated lymphoid tissue lymphoma in the bilateral upper lung lobes. ^18^F‐fluorodeoxyglucose (FDG)‐positron emission tomography‐computed tomography scans were performed for systemic evaluation. In addition to FDG accumulation in the lung lesion, focal accumulation (SUVmax 4.30) was found in the small intestine in the pelvic region of the left lower abdomen, where circumferential wall thickening was observed (Figure 1). Because a small intestinal tumor or inflammatory lesion was suspected, the patient was referred to our hospital.

**FIGURE 2:**
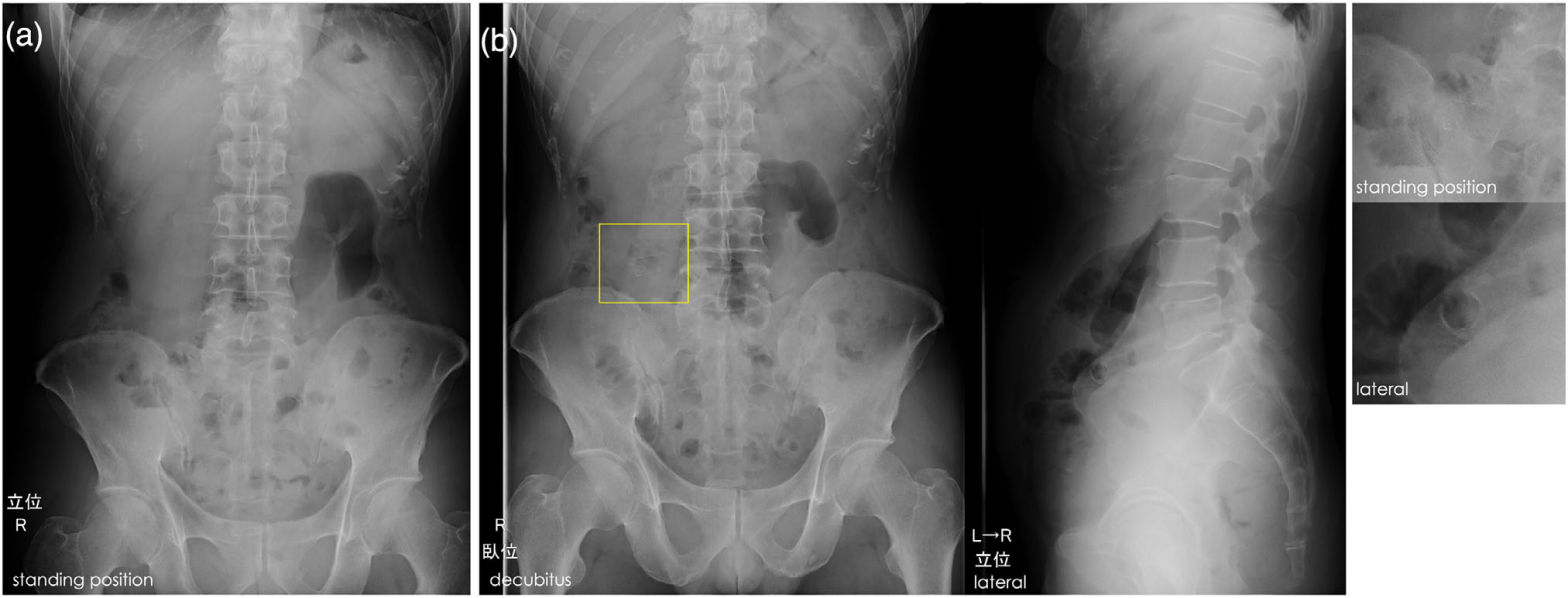
Plain abdominal X‐ray imaging. (a) Plain abdominal radiography (standing position) showed no patency capsule or shape deformity. (b) Plain abdominal radiographs (lateral and decubitus positions) showing the localization of the patency capsule (PC, in the lateral and decubitus views. The PC had already dissolved into a shell only (yellow box).

Computed tomography (CT) showed wall thickening in the pelvic region of the left lower abdomen (Figure 1). We planned to SBCE examination. On the day before SBCE, the patient fasted for 12 h and then swallowed a PC. No obvious symptoms of abdominal pain or bowel obstruction were present after PC administration. One bowel movement was observed after PC administration; however, PC discharge was not visually confirmed during the 24‐h period. Therefore, plain abdominal radiography was performed to clarify the PC localization. No obvious PC was observed on standing plain abdominal radiography (Figure 2a). Since the patient had also had bowel movements the day before, we concluded that the PC had been discharged inconspicuously, and judged that the patient had intestinal patency. Therefore, SBCE was initiated approximately 24 h after the PC was administered.

**FIGURE 3:**
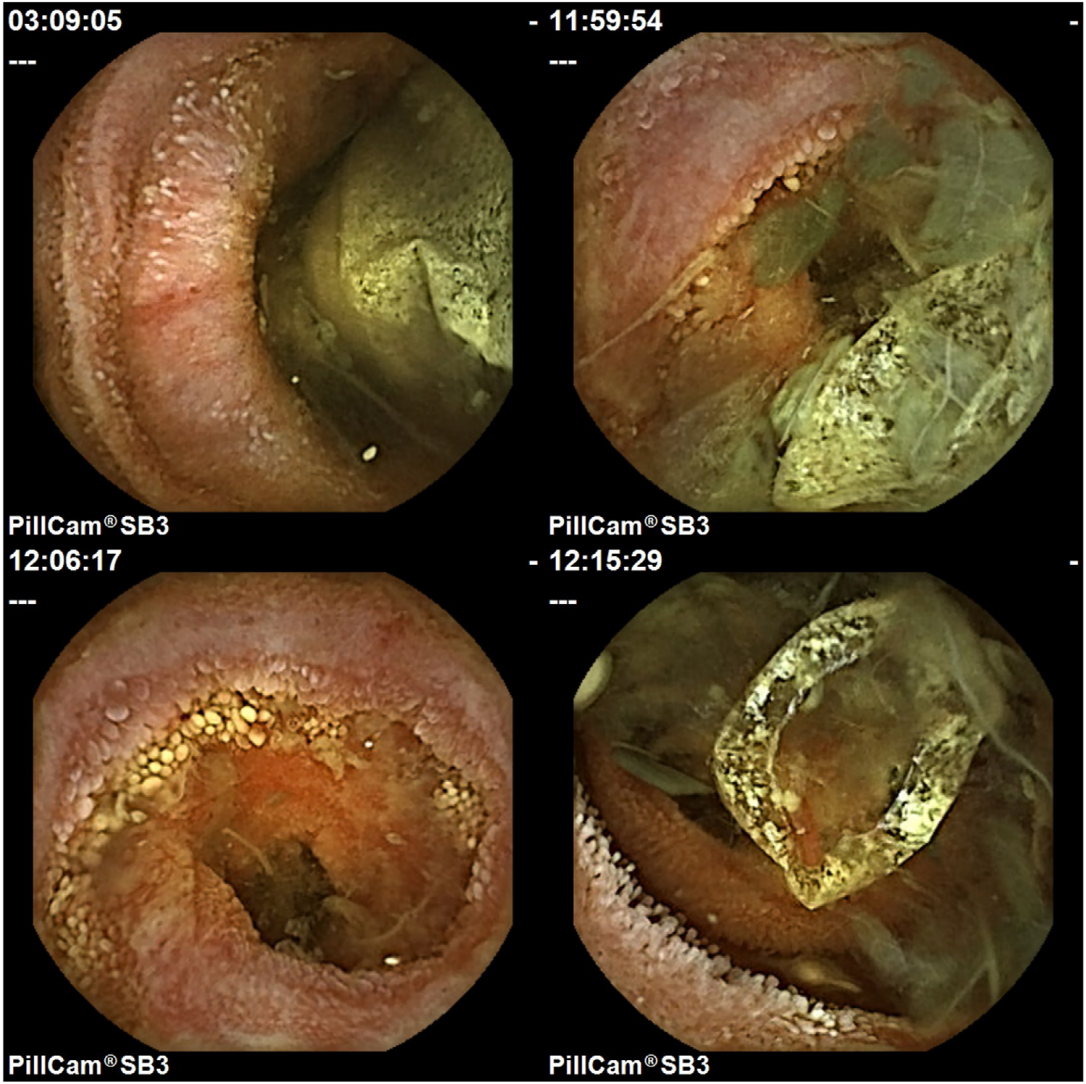
Small bowel capsule endoscopy (SBCE) findings. SBCE revealed a neoplastic lesion with stenosis and a patency capsule shell on the oral side of the stenosis.

However, SBCE captured a neoplastic lesion with stenosis at a site thought to be in the upper small intestine and then remained static at that site throughout the battery runtime. Furthermore, SBCE revealed the PC shell in the intestinal tract on the oral side of the stenosis (Figure 3). We revisited the plain abdominal radiographs taken earlier, and upon enlargement of the lateral and decubitus view images, we found that the PC had already dissolved to only a shell. Reviewing the standing view again, we could barely recognize the shell overlapping the sacrum (Figure 2a,b).

**FIGURE 4:**
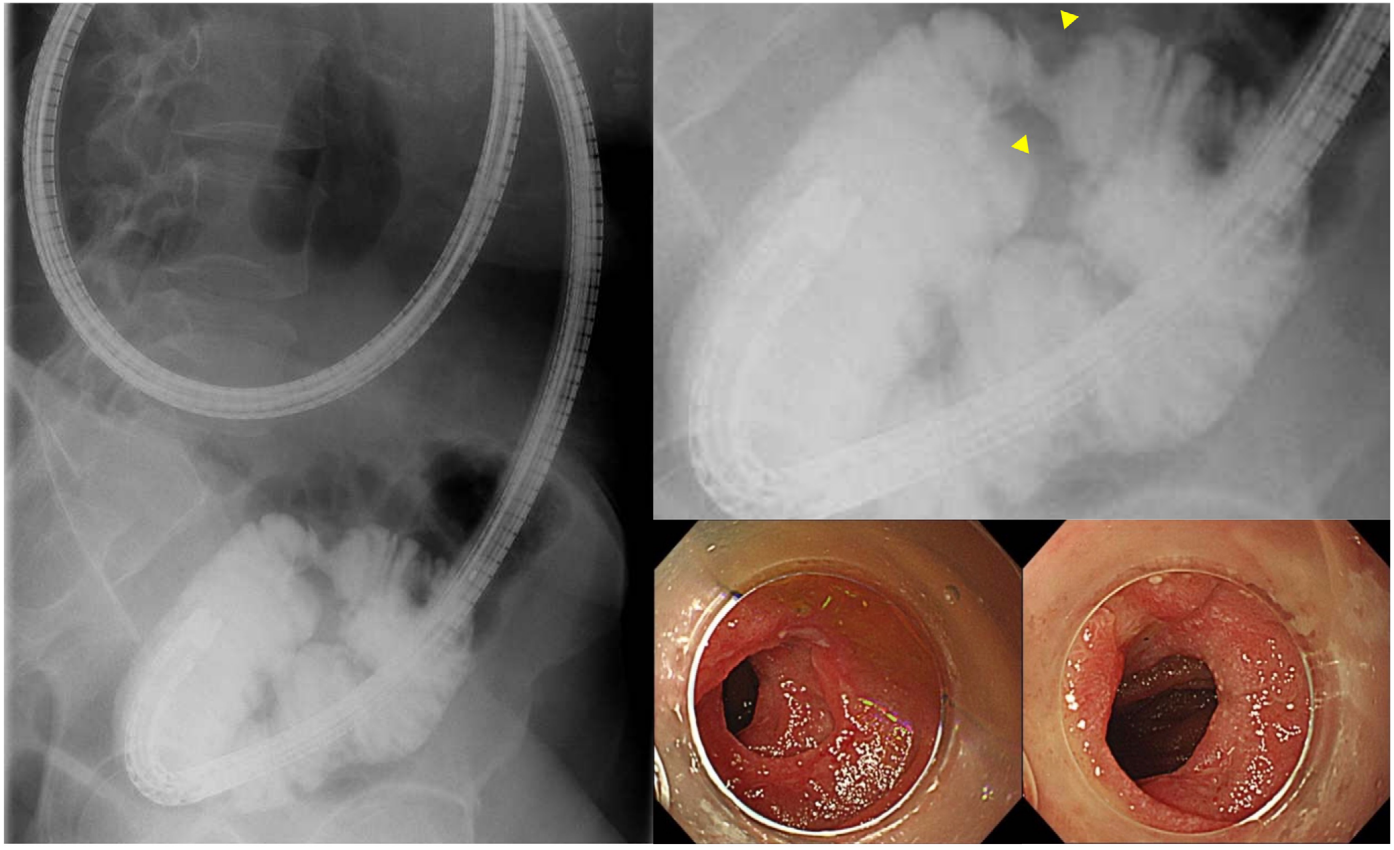
Balloon‐assisted enteroscopy. A neoplastic lesion with stenosis and loss of villous structure is observed in the jejunum, approximately 70 cm from the ligament of Treitz (yellow arrowhead). The patency capsule shell and small bowel capsule endoscopy passed at the time of the examination.

Since the PC shell was lodged and the neoplastic lesion imaged by the SBCE was highly stenotic, the SBCE could be retained in the small intestine for >2 weeks. Therefore, transoral balloon‐assisted enteroscopy (BAE) was performed 7 days post‐SBCE with a view to capsule retrieval and qualitative diagnosis of the upper small intestine neoplastic lesion. A neoplastic lesion with stenosis and loss of villous structure was observed in the jejunum, approximately 70 cm from the ligament of Treitz (Figure 4). However, the SBCE and PC shell were not present on the oral side of the stenosis at that time and were also not seen on fluoroscopic images. We considered that the PC shell and SBCE may have squeezed through the stenosis during the intervening 7 days. A biopsy of the neoplastic lesion was diagnosed as a small intestinal lesion related to the previously diagnosed mucosa‐associated lymphoid tissue lymphoma.

## DISCUSSION

If a small intestinal tumor is clearly localized on CT and can be reached with a single insertion, BAE is performed without prior SBCE examination. However, since it is difficult to determine the insertion route for balloon small bowel endoscopy in the case of small intestinal tumors in the pelvic cavity, SBCE screening is performed. For SBCE in cases with diseases associated with small bowel stenosis, a PC is used to assess gastrointestinal patency to avoid the risk of capsule endoscope retention by the stenosis.^2^ Unlike Western PCs (Agile PC), PCs without radiofrequency identification tags are distributed in Japan.^2^ The basic performance of the PC remains the same: 30 h after administration, intestinal fluid enters the PC from timer plugs on both sides and it begins to dissolve. After approximately 5 days, the PC disintegrates, leaving only the parylene film shell. If the PC did not change its shape and could be visually confirmed to be discharged from the body within 30–33 h of administration, intestinal patency is assumed. Additionally, if the PC is seen to have reached the large intestine on radiography, low‐dose CT, tomosynthesis, or abdominal ultrasound, the patency of the small intestine can be accepted.^3^, ^4^, ^5^ It has been reported that, if the PC is not depicted on plain radiographs, it may have been inconspicuously discharged from the body, and SBCE can be performed without major adverse events.^6^ However, it is difficult to localize the PC in the body on plain radiographs, and judgment errors may occur,^2^ which can result in SBCE retention.

In our case, the PC had completely dissolved within 24 h of administration. To the best of our knowledge, no previous case of complete dissolution of a PC, leaving only its shell during a 30‐h patency evaluation period. The usefulness of determination at the 24‐h post‐PC administration time point has been previously reported by us.^7^ The battery life of the SBCE is approximately 15 h. If a patency capsule was present in the small intestine 24 h after the patency capsule was taken, observation of the entire intestinal tract by SBCE could be difficult. Therefore, the patency evaluation decision was made after 24 h. The undissolved PC shell appears as an opaque shape on plain abdominal X‐ray images because of the barium contained in the PC. Out‐of‐spec variations in PC dissolution time may be caused by too much or too little exposure to intestinal fluids, or by chewing the PC during ingestion. Some case reports have described a lack of PC dissolution because of low exposure to intestinal fluids.^8^ In our case, the patient took Patency capsules at home after careful explanation and consent. Although medical personnel could not be present, the patient was a middle‐aged man without dementia with no problematic behavior regarding swallowing the Patency capsules, such as chewing the PCs. Plain abdominal radiography within 30‐h post‐ingestion showed no opaque PC, but the PC might have remained in the body because of premature dissolution caused by high exposure to intestinal fluid. In addition, early dissolution within 30 h in Agile PC is reported to be at a rate of 1.3%, suggesting that it may be influenced by the quality of the PC itself.^9^

As a follow‐up evaluation, we referred the PC patency evaluation to two capsule endoscopy specialists. We presented them with only the standing radiographic images obtained 24 h after PC ingestion. Based on their experience, they also judged that the intestines were patent. Thus, it is not common for a PC to dissolve completely leaving only the shell during the 30‐h evaluation of patency, and it may be difficult to make an accurate determination without a thorough evaluation of the images. Looking ahead, we believe that technological support, such as diagnostic AI assistance in analyzing images, is an area that can be expected.

In conclusion, evaluation of intestinal patency by PC is important for safer SBCE in patients with suspected stenosis. However, if the expulsion of the PC is not visually confirmed, internal remnants may be present due to premature dissolution of the PC, which may need to be confirmed by using other modalities or using plain abdominal radiography without preconceived notions that the PC will not dissolve within 30 h of administration.

## CONFLICT OF INTEREST STATEMENT

None.
